# Complementary Cell Lines for Protease Gene-Deleted Single-Cycle Adenovirus Vectors

**DOI:** 10.3390/cells12040619

**Published:** 2023-02-14

**Authors:** Seyyed Mehdy Elahi, Nazila Nazemi-Moghaddam, Claire Guilbault, Mélanie Simoneau, Rénald Gilbert

**Affiliations:** 1 Department of Production Platforms & Analytics, National Research Council Canada, Building Montreal, Montreal, QC H4P 2R2, Canada; 2 Department of Bioengineering, McGill University, Montreal, QC H3A 0E9, Canada

**Keywords:** protease-deleted adenovirus, single-cycle adenovirus vector, stable cell line, suspension culture

## Abstract

To increase the safety of adenovirus vector (AdV)-based therapy without reducing its efficacy, a single-cycle adenovirus vector (SC-AdV) with a deletion in the protease gene (PS) was developed in order to be used as a substitute for the replication-competent adenovirus (RC-AdV). Since no infectious viral particles are assembled, there is no risk of viral shedding. The complementary cell lines for this developed AdV proved to be suboptimal for the production of viral particles and require the presence of fetal bovine serum (FBS) to grow. In the current study, we produced both stable pools and clones using adherent and suspension cells expressing the PS gene. The best adherent cell pool can be used in the early stages for the generation of protease-deleted adenovirus, plaque purification, and titration. Using this, we produced over 3400 infectious viral particles per cell. Additionally, the best suspension subclone that was cultured in the absence of FBS yielded over 4000 infectious viral particles per cell. Harvesting time, culture media, and concentration of the inducer for the best suspension subclone were further characterized. With these two types of stable cells (pool and subclone), we successfully improved the titer of protease-deleted adenovirus in adherent and suspension cultures and eliminated the need for FBS during the scale-up production. Eight lots of SC-AdV were produced in the best suspension subclone at a scale of 2 to 8.2 L. The viral and infectious particle titers were influenced by the virus backbone and expressed transgene.

## 1. Introduction

One of the most commonly used adenovirus vectors (AdV) in research and development is the replication-defective adenovirus (RD-AdV). Because the E1 region of RD-AdV is deleted, it needs to be grown in a complementary cell line, such as HEK293A [1]. Usually, in RD-AdVs, the E3 region is also deleted in order to increase the cargo capacity by up to 8 kb. For applications of AdV in cancer therapy and vaccination, amplification of the therapeutic gene expression is desirable (for a review, see [2,3,4,5,6]). In contrast to RD-AdV, in replication-competent AdV (RC-AdV), the transgene DNA is replicated and can express the transgene more abundantly [7,8]. However, there is concern about the safety of RC-AdV in vivo in humans because of its capacity to replicate and form infectious particles. Single-cycle AdV (SC-AdV) is considered a safer alternative that, compared to RD-AdV, can provide higher levels of transgene expression. The SC-AdVs contain the necessary genes for DNA replication but are deleted of a viral late gene involved in the production of progeny viruses, such as IIIa [8] or protease [9]. The SC-AdVs replicate their genomes and transgenes as well as RC-AdV but generate more robust and persistent immune responses against the transgene compared to RD-AdV or RC-AdV [7].

Previously, we developed a SC-AdV based on the deletion of one of the essential late viral genes, i.e., the protease (PS) [9]. The ability of protease-deleted AdV to undergo a single round of replication within the host cell led to a higher expression level of the therapeutic gene compared to RD-AdV ([10,11,12] without the risk of viral shedding. The replication of a protease-deleted AdV is possible only in a cell line if the protease is provided in trans.

Long-term expression of the gene of interest (GOI) is desirable in many applications and could be achieved by different methods, such as the random integration of GOI into the cell genome following the transfection of cells with plasmid; transduction of cells with a lentivirus vector; sleeping beauty transposon system; or newer technologies, such as Tal effector nucleases (TALENs) and clustered regularly interspaced short palindromic repeats (CRISPRs). Each method has its own benefits and drawbacks [13,14,15,16,17,18,19,20,21,22].

In a stable cell line, the GOI expression can be under the control of either a constitutive or an inducible promoter. To regulate both the level and the duration of the expression of a gene whose constitutive expression might not be tolerated by the cell, such as the adenovirus PS, an inducible expression system, such as the “cumate gene switch” [23], could be used. In the repressor configuration of the cumate gene switch, regulation is mediated by the binding of the repressor (CymR) to the operator site (CuO), placed downstream of a strong constitutive promoter, such as CMV. The resulting promoter is known as CMV5-CuO. The addition of cumate (4-isopropylbenzoic acid, Sigma–Aldrich, St-Louis, MO, USA) relieves the repression. In the activator configuration, a chimeric transactivator “cTA” can activate the transcription from a minimal CMV promoter placed upstream of multiple copies of the operator site in the absence of the cumate. The resulting promoter is known as CR5. Upon the introduction of cumate, the activator can no longer bind DNA and, therefore, is unable to activate the transcription from the basal promoter. To express a transgene regulated by CR5, the cTA is provided by a stable cell line expressing cTA. In the current study, we used both strategies to control the PS expression. We also used the coumermycin/novobiocin-regulated gene expression system [24] for controlling the PS expression. In this inducible expression system, in the presence of the coumermycin, the chimeric transactivator (λR-GyrB) is dimerized and binds to the lambda operator (λOP) to turn on the transcription of PS. The expression of λR-GyrB is also under its own regulation. In the absence of coumermycin, only a very low basal expression level of the transactivator occurs.

Previously, we generated a few stable cell lines expressing the PS gene of adenovirus type 2 [9,25]. The two most successful stable cell lines were 293rTA-PS#7 and HEK293-CB6. In the case of 293rtTA-PS#7, the PS gene was expressed from a tetracycline-inducible promoter in a dicistronic vector co-expressing the green fluorescent protein (GFP). The presence of 5% serum was needed for the cell line to grow either in adherence or suspension. Although this cell line allowed us to produce many stocks of protease-deleted AdVs, there were a few limitations: (I) Overexpression of PS was deleterious to the cells and its expression needed to be kept in the OFF condition, in the absence of the inducer. When PS was induced, the yield of the PS-deleted virus was reduced about five-fold [9]. The PS toxicity prevented the co-expression of any other genes by the cell line using the same gene switch. (II) The presence of FBS in the culture media is challenging for clinical applications due to its complex and variable composition and the risk of contamination with animal viruses. (III) Finally, the co-expression of GFP in the cell line interfered with the screening and titration of AdVs with GFP as their GOI. Another cell line, the HEK293-CB6, expressing the adenovirus type 2 PS under the control of a heat shock element was developed for enabling plaque purification and titration of Ps-deleted AdV [25]. Unfortunately, the titer of a wild type AdV deleted of the PS gene (AdPS-) was more than 100 times lower compared to an AdV with an intact PS gene in the HEK293A cell line. For the successful advancement and application of our PS-deleted adenovirus platform for cancer therapy or the development of vaccines, improved versions of both adherent and suspension PS-complementary cell lines that provide higher viral yields were needed. In addition, the cells should be adapted to suspension culture using an animal component-free medium to facilitate the scale-up and approval of regulatory agencies.

This study describes the development of stable pools and subclones that express the PS gene under the control of different promoters. The stable pools and subclones were screened for the production of a wild type and/or recombinant PS-deleted AdV expressing a toxic gene. The two best cell lineages—one adherent pool and one subclone—adapted to the suspension culture and were selected as candidates for the isolation and initial growth of PS-deleted AdV and its subsequent scale-up, respectively. These two selected cell candidates can greatly facilitate our capacity for the isolation as well as large-scale production of PS-deleted AdV for gene and cancer therapy applications.

## 2. Materials and Methods

### 2.1. Cells Culture Conditions and Viruses

Two stable anchorage-dependent cell lines; HEK293A [1] (obtained from ATCC), and HEK293A-cTA [23] (produced in-house) were grown in Dulbecco’s modified Eagle’s medium (Gibco, Life Technologies, Ottawa, ON, Canada), supplemented with 5% of FBS (Hyclone, Logan, TU, USA) and 2 mM of glutamine (Gibco, Life Technologies, Ottawa, ON, Canada). Two suspension culture cell lines, i.e., 293S (Gibco, Life Technologies, Ottawa, ON, Canada) and SF-BMAdR [26] (produced in-house), were grown in SFM4Transfx-293 medium (SFM4-T, Hyclone, Logan, TU, USA) and PRO293s CDM (PRO293s, Lonza, Walkersville, MD, USA) was supplemented with 6 mM of glutamine, respectively. The SF-BMAdR cell line is a modified A549 cell line that expresses the E1 gene of the adenovirus and was adapted to culture in suspension- and serum-free media [26]. All of the generated pools, clones, and subclones were grown in the same medium as their parental cell line. Adherent cells were grown in treated 10 and 15 cm plates (Sarstedt, Numbrecht, North Rhine-Westphalia, Germany). Depending on cell culture volume, the suspension cultures were performed in 96-, 24-, and 6-well plates (Sarstedt, Numbrecht, North Rhine-Westphalia, Germany), or in 125 mL shake flasks (Corning, Oneonta, NY, USA). Long-term cultivation for the stability study and cell banking was conducted in 100 mm dishes or 125 mL shake flasks. Suspension culture agitation was conducted at 100–120 rpm using orbital shakers (Bellco, or GL-300 Analytics). Cell banks of frozen vials were made in a culture medium supplemented with 7.5% DMSO (Sigma–Aldrich, St-Louis, MO, USA) and kept frozen in liquid nitrogen. All cells were cultivated at 37 °C and 5% CO_2_.

The AdPS- (a wild type AdV deleted of the protease gene) [9] and AdPS-/CU, a PS-, E1-, and E3-deleted adenovirus, which expresses the CD::UPRT fusion gene (cytosine deaminase::uracil phosphoribosyl transferase) in a dicistronic cassette with GFP [10], were used for screening pools and clones expressing the PS gene.

To show the efficacy of our selected suspension cell line, in addition to the above viruses, four other viruses were produced at larger scales: AdPS-/HCV-NS3-5a [11] is a SC-AdV with deletions in the PS, E1, E3, and E4 (except ORF6) regions, which expresses the NS3 to NS5a proteins of HCV in a dicistronic cassette with the adenovirus E1a gene; AdPS-/Her2 ([12] is a SC-AdV with deletions in the PS, E1, and E3 regions, which expresses the rat HER2/neu (human epidermal growth factor-like receptor) gene in a dicistronic cassette with the adenovirus E1a gene; AdPS-/TRP2-IRES-GM_CSF and AdPS-/GP100-IRES-GM_CSF are both SC-AdVs with deletions in the PS and E4 (except ORF6) regions. The AdPS-/TRP2-IRES-GM_CSF expresses the tyrosinase-related protein-2 (TRP2) and granulocyte-macrophage colony-stimulating factor (GM-CSF); AdPS-/GP100-IRES-GM_CSF expresses a melanoma-associated antigen (GP-100) and GM_CSF. The expression of GM_CSF is in both viruses, being under the translational control of an IRES (internal ribosome entry site).

The original backbone plasmids for all of the lentiviruses used in this study were based on pCSII-CMV-mcs in which the Tet07 promoter was introduced into the 3′-long terminal repeat [27], and the CMV promoter for the expression of the adenovirus protease was replaced by either the inducible CMV5-CuO or CR5 promoters [23,28]. The adenovirus PS gene was amplified by the polymerase chain reaction over pAdTR5-PS-GFP [29] and was subcloned into the XbaI site of two lentivirus vectors to generate Tet07-CSII-CMV5-Cuo-PS and Tet07-CSII-CR5-PS. The repressor CymR gene [23] was cloned in Tet07-CSII-CMV5 to generate Tet07-CSII-CMV5-CymR.

The puromycin gene under the control of the SV40 promoter was excised from pPURO-25 (Clontech, Mountain View, CA, USA) by digesting with PvuI and BamHI and was cloned in the NdeI site of pGyrB [24]. A fragment that contained 12 copies of the λ operator (12λop), TATA box, and the protease gene was synthesized by GeneArt Gene Synthesis and cloned in the NaeI site of pGyrB-PURO, resulting in pλR-GyrB-PS, which expresses PS under the control of the inducible coumermycin promoter and also expresses puromycin as an antibiotic resistance gene.

Three lentiviruses, i.e., Lenti-CMV5-Cuo-PS, Lenti-CR5-PS, and Lenti-CMV5-CymR, were produced by transient transfection of pTet07-CSII-CMV5-Cuo-PS, pTet07-CSII-CR5-PS, and pTet07-CSII-CymR in the 293SF-PacLV cell line, respectively [27]. The protocols for transfection and concentration of lentivirus were previously described in detail [30]. Briefly, for the generation of each lentivirus, two 150 mm dishes of 293SF-PacLV were transfected with 45 µg of the appropriate plasmid. Doxycycline (1 μg/mL, Sigma–Aldrich, St-Louis, MO, USA) and cumate (50 μg/mL) were added at 4–6 h post-transfection to induce the expression of the LV components of packaging cell lines. The supernatant was harvested at 48 and 72 h post-transfection and concentrated 40 times by centrifugation at 100,000× *g* for 2 h at 4 °C. The LV pellet was resuspended overnight in 1 mL of culture medium containing 1% FBS at 4 °C and then aliquoted and stored at −80 °C until use.

#### Generation of Stable Cell Lines by Lentiviruses

To generate each stable cell line, 200 μL of each concentrated lentivirus was incubated with 8 μg/mL of polybrene (Sigma–Aldrich, St-Louis, MO, USA) and incubated for 30 min at 37 °C. After the incubation period, 150,000 parental cells in 24-well plates were transduced by the appropriate lentivirus. To increase the transgene copy number, after one or two passages (which were needed to augment the cell number and allow for cell recovery after transduction), a fraction of the cells were transduced again and this process was repeated up to four times. To generate HEK293A-CymR-PS and HEK293A-cTA-PS, the HEK293A-CymR and HEK293A-cTA cell lines were transduced up to two times with Lenti-CMV5-Cuo-PS and Lenti-CR5-PS, respectively. To generate the 293S-CymR-PS and SF-BMAdR-CymR-PS cell lines, the 293S cell line and the SF-BMAdR cell line were transduced first with Lenti-CMV5-Cuo-PS (to obtain a cell line without the CymR repressor), followed by two-time transduction with Lenti-CMV5-CymR (to obtain a cell line in which the expression of PS was under the control of the CymR repressor). Finally, cells were transfected two more times with Lenti-CMV5-Cuo-PS (total of 5 transductions) to increase the expression level of the PS gene. After each transduction, a part of the new cell lineage was kept in the culture separately to test for the production of AdPS- after the scale-up production. The SF-BMAdR-CymR-PS pool was subcloned by limiting the dilution as described previously [26]. The clones were screened for the production of AdPS-. Briefly, 750,000–1,000,000 cells from each cell line (pool, clones, or subclones) were infected in 6-well plates with AdPS- with a MOI of 5 TCID_50_. Cumate, if needed, was added at a concentration of 50 μg/mL at 5 h post-infection (hpi) to initiate the expression of PS by the cells. At 48 hpi, the infected cells were harvested and refrozen–thawed 3 times. As AdPS- does not express any cell marker (such as GFP), the virus titer was measured with a TCID_50_ assay on HEK293A-CymR-PS cells. Briefly, 96-well plates were seeded at a concentration of 15,000 cells/well. The next day, the medium was replaced with 100 μL of 10-fold serial dilution of viral inoculum. Six wells were infected for each dilution. Wells that were positive for the cytopathic effect were recorded after 7–9 days; the TCID_50_ titer was calculated using the Spearman–Karber method [31] and expressed as TCID_50_/mL.

### 2.2. Generation of the SF-BMAdR-λR-GyrB-PS Cell Line by Transfection

A subclone of the SF-BMAdR cell line was transfected with pλR-GyrB-PS under the puromycin selection and cloned in 96-well plates, as described previously, for the generation of the parental cell line [26]. Briefly, the SF-BMAdR-λR-GyrB-PS cells were generated by co-transfecting 10 million cells maintained in SFM4-T with 50 μg of pλR-GyrB-PS (linearized with PvuI) using lipofectamine 2000 (Invitrogen, Carlsbad, CA, USA), according to the manufacturer’s recommendation. Moreover, 48 h after transfection, the cells were diluted in 96-well plates (5000 and 10,000 cells/well) in the presence of 0.625 μg/mL of puromycin (Sigma-Aldrich, St-Louis, MO, USA). Resistant colonies were isolated and expanded in the same medium. The clones were screened for the production of AdPS-/CU. Briefly, the cells were plated in 96-well plates at 50,000 cells/well. They were infected at a MOI of 5 transducing units (TU)/cells with AdPS-/CU in the presence or absence of 50 μg/mL cumate plus 5 nM coumermycin A1 (Sigma-Aldrich, St-Louis, MO, USA) to induce the expression of the PS gene. Adding the cumate was needed to confirm the clones, which were derived from SF-BMAdR cells, and still expressed the CymR gene since the titer would be higher in the absence of cumate because of the toxicity of CU. However, the expression of the PS gene only depends on the presence of coumermycin. At 24, 48, and 72 hpi, the plates were scanned on a fluorescence imaging system (Typhoon Trio + scanner; Amersham Biosciences, GE Healthcare, Chicago, IL, USA); for each clone, the relative fluorescence intensity in the presence or absence of cumate (ON/OFF ratio) was computed to isolate the clones that were still expressing CymR. To estimate the AdV productivity, the cells were harvested at 72 hpi and lysed by three freeze/thaw cycles. Dilutions of lysate were applied on HEK293A cells in 96-well plates. Moreover, 72 h later, the plates were scanned on a fluorescence imager and the intensity of the signal was computed. For the best clones, the infectious titer of the AdV (TU/mL) was determined by measuring the percentages of GFP-positive cells after infection with HEK293A by flow cytometry using an LSR II (BD Biosciences, Mississauga, ON, Canada) or a FC 500 MPL (Beckman-Coulter, Mississauga, ON, Canada) flow cytometer [32]. Subcloning was performed by limiting dilution. The subclones were picked and expanded in a PRO293s medium. They were then tested for the production of AdPS-/CU as described above. The stabilities of the 7 best subclones of SF-BMAdR-λGyrB-PS and HEK-293A-CymR-PS pool for the production of AdPS-/CU were evaluated over a period of two months. To have comparable data of the virus progeny, the best SF-BMAdR-λGyrB-PS subclone and the HEK293A-CymR-PS pool were compared in parallel by the TCID_50_ assay over infected cells with AdPS-, with a MOI of 5 at 48 hpi. The best harvesting time for the SF-BMAdR-λGyrB-PS cell line was determined by evaluating the virus growth curve up to 96 hpi. Briefly, two shaker flasks of SF-BMAdR-λGyrB-PS cells with 25 mL working volume and a concentration of 500,000 cells/mL were infected with AdPS- with a MOI of 5. Coumermycin was added at a concentration of 5 nM at 5 hpi to induce the expression of the PS gene. Samples were taken at 5, 24, 48, 72, and 96 hpi, and after 3 freeze/thaw cycles, they were titrated by the TCID_50_ assay in duplicate.

As overexpression of the PS gene is deleterious for virus progeny; the optimal concentration of coumermycin for the production of AdPS- was evaluated in the BMAdR-SF-PS-174-12A subclone. Briefly, the wells of a 6-well plate at a concentration of 500,000 cells/mL were infected with a MOI of 5 TCID_50_ in duplicate. At 5 hpi, coumermycin was added at concentrations of 0.2, 0.5, 1, 2.5, and 5 nM, and 2 infected wells were left as non-induced. The specific productivity was determined by the TCID_50_ assay in duplicate at 48 hpi.

Data were analyzed using GraphPad Prism (GraphPad^®^ Software, San Diego, CA, USA). Statistical significance of the difference between groups was calculated by a 1-way analysis of variance (ANOVA) with Tukey’s multiple comparison test or unpaired *t*-test. Significance was considered non-significant (ns) for *p* > 0.05 and significant as * for *p* values < 0.05, ** for *p* values < 0.01, and *** for *p* values < 0.001.

### 2.3. Production and Purification of SC-AdVs in the SF-BMAdR-λR-GyrB-PS-174-12A Cell Line at a Larger Scale

The SF-BMAdR-λR-GyrB-PS-174-12A cells were cultured in PRO293s CDM supplemented with 6 mM of glutamine and scaled up to 2 L in Corning shake flasks under the same conditions as the small-scale production. During the cell culture scale-up, cell concentration was kept between 2.5 × 10^5^ and 1.5 × 10^6^ cells/mL. On the day of infection, cells were diluted with fresh medium (between 4.9 to 5.5 × 10^5^ cells/mL). The total volume of infected cells varied (depending on our need) between 2 and 8.2 L. The cell culture was divided into 2 L shaker flasks with a working volume of ≅500 mL/flask. Cells were infected with 5–10 infectious particles with each seed stock depending on the volume of infection and the titer of the seed stock. Coumermycin was added at a concentration of 20 nM at 5 hpi. Three days post-infection, cells were centrifuged and cell pellets were resuspended in 20–50 mL of conditioned medium and lysed by three freeze/thaw cycles. For virus purification, the supernatant of the cell lysis was purified by CsCl two-step-gradient (1.2 and 1.4 g/cm^3^) centrifugation (59,000× *g*, 4 °C, 1 h 30) followed by continuous CsCl gradient centrifugation (59,000× *g*, 4 °C, 20 h). Finally, the upper band of the gradient was collected and the buffer was exchanged using a NAP 25 desalting column (GE Healthcare, Buckinghamshire, IJ, UK). Purified viruses were frozen in 50 mM of Hepes pH 7.5, 150 mM NaCl, 2 mM MgCl_2_, and 5% sucrose. For quantification of the viral particles (VP)/mL, the virus was diluted in a lysis solution (0.1% sodium dodecyl sulfate (SDS), 10 mM Tris-Cl pH 7.4, 1 mM EDTA)) for 10 min at 56 °C. The OD260 was read using the Nanodrop spectrophotometer and VP/mL was calculated by multiplying the OD by the dilution factor and the extinction coefficient of 1.1 × 10^12^ [33]. The infectious titer was measured using the “Adeno-X™ Rapid Titer Kit” (Clontech Laboratories, Inc., Mountain View, CA, USA). Briefly, HEK293A cells were infected with serial dilutions of AdV, fixed after 2 days, incubated with a hexon protein-specific antibody, and followed by incubation with a secondary antibody conjugated to horseradish peroxidase. The readout was performed by adding a DAB precipitating substrate and manually counting positive cells under a brightfield microscope. The titer was expressed as infectious units (ifu)/mL.

## 3. Results

### 3.1. Generation of a Stable Cell Line by Lentiviruses

Four lineages of cell pools were produced using lentiviruses. Each parental cell line was transduced up to five times with one or two lentiviruses. After each transduction, a fraction of the transduced cells was kept in the culture and another fraction was transduced again. Finally, all of the pools were tested for AdPS- production. The specific productivity of AdPS- was calculated for each pool. The expression of the PS gene in each cell line was under the control of “the cumate gene switch” (Table 1). In adherent HEK293A-CymR-PS, suspension 293S-CymR-PS, and BMAdR-SF-CymR-PS the expression of the PS gene was under the control of the repressor configuration of the switch. Cumate was added after infection to induce the expression of the PS gene. However, in the case of the adherent HEK293A-cTA-PS cell line, the expression of the PS gene was under the control of the transactivator of the switch. Cumate was present during the cell culture to prevent the binding of cTA (transactivator) to the operator binding sites and, consequently, block the expression of the PS gene. At the time of infection, cumate was removed by the medium replacement. In the absence of cumate (ON condition), cTA activates the transcription of the PS gene.

In the case of the HEK293A-CymR-PS lineage, only one time transduction with Lenti-CMV5-Cuo-PS was enough for AdPS- production at a high titer (Table 1); a second transduction had deleterious effects on the virus titer. However, in the case of the HEK293A-cTA-PS lineage, a second transduction actually improved the titer.

In 293S-CymR-PS and SF-BMAdR-CymR-PS stable pools, the expression of PS was controlled by the presence of CymR after two transductions with lentiviruses expressing CymR (Lenti-CMV5-CymR). There is a direct correlation between the number of transductions with Lenti-CMV5-Cuo-PS and AdPS- titer when virus progeny was measured in the presence of the cumate (ON condition). The titer of AdPS- increased by 26 and 51 times after 3 transductions with the Lenti-CMV5-Cuo-PS in SF-BMAdR-CymR-PS and 293S-CymR-PS pools, respectively (Table 1).

Between the two adherent pools, HEK293A-CymR-PS produced more infectious particles than HEK293A-cTA-PS. In addition, working with this pool was easier as cumate was added directly to the medium after infection, contrary to HEK293A-cTA-PS, which required removing the culture medium and washing the cells before infection to remove all traces of the cumate. We did not see the need to clone our pool in order to improve the titer because the HEK293A-CymR-PS cells were only needed at early stages for the generation of recombinant AdV, plaque purification, and the quantification of infectious particles of PS-deleted AdV by TCID_50_ or the plaque assay.

Despite a slightly lower titer obtained by the BMAdR-SF-CymR-PS pool, at 3000 TCID_50_/cell compared to 4335 for 293S-CymR-PS, we chose BMAdR-SF-CymR-PS for cloning by limiting the dilution since the use of this cell line would reduce the risk of recombination and the formation of replication-competent adenovirus (RCA) when working with SC-AdV, carrying a gene of interest in the E1 region [10].

**Figure 1:**
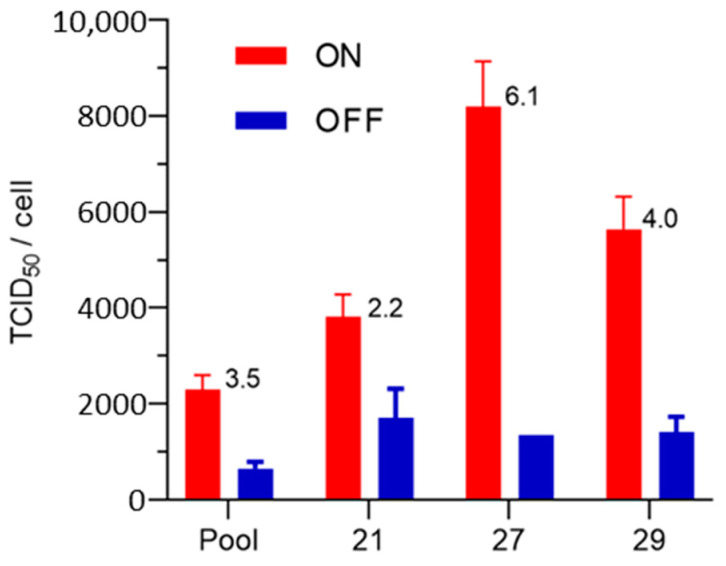
Production of AdPS- by the SF-BMAdR-CymR-PS pool and the 3 best clones (21, 27, and 29). Two wells of six-well plates for each subclone were infected with AdPS- using a MOI of 5 TCID_50_/cells in the presence or absence of the cumate (*n* = 2). The specific productivity was measured by TCID_50_ in duplicate and the ON/OFF ratio values are indicated above the bars. The data represent the mean ± SEM.

The pool of SF-BMAdR-CymR-PS was subcloned by limiting dilution in 96-well plates. The production of AdPS- was measured in 17 subclones and pools under ON and OFF conditions (with or without the addition of the cumate), as shown in the Supplementary Materials (Figure S1). The majority of the clones produced less than 10 particles per cell. Only four clones were superior to the pool in the AdPS- production. The experiment was repeated in duplicate for the pool and the three best clones (Figure 1). The best clone (SF-BMAdR-CymR-PS#27) produced three times more adenovirus per cell than its pool.

### 3.2. Generation of the SF-BMAdR-λR-GyrB-PS Cell Line by Transfection

**Figure 2:**
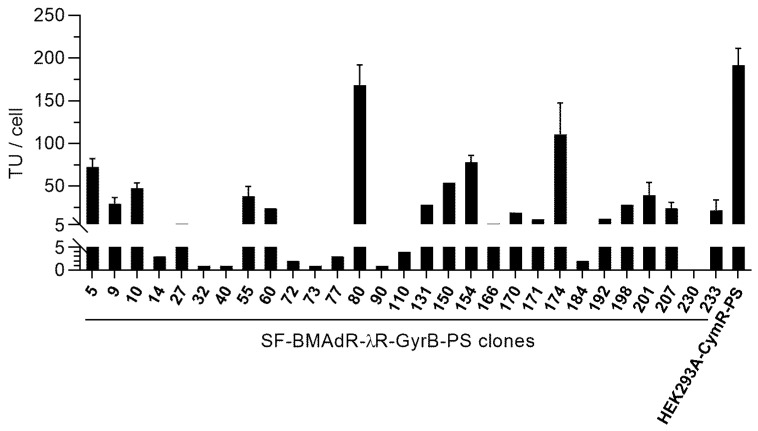
Comparative production of AdPS-/CU by SF-BMAdR-λR-GyrB-PS clones. Cells were infected with AdPS-/CU using a MOI of 5 transduction unit(s) (TU)/cell in the presence of coumermycin (5 nM) for SF-BMAdR-λR-GyrB-PS clones and cumate (50 μg/mL) for HEK293A-CymR-PS pool. The specific productivities of infectious vector particles (TU/cell) were determined by flow cytometry and expressed as TU/cell. The samples with error bars were tested in triplicate (*n* = 3) and are presented as mean ± SEM. The samples without error bars were analyzed only once (*n* = 1).

The SF-BMAdR-λR-GyrB-PS cell line was generated by the standard transfection method. The transfected cells were cloned at 2 days post-transfection. Over 240 clones were analyzed by imaging; from those, 29 were tested for the production of AdPS-/CU. As the expression of the PS gene in the cell line is under the coumermycin/novobiocin-regulated gene expression system, screening of clones was performed in the presence of 5 nM of coumermycin (ON condition). We did not analyze clones in the OFF condition because the analysis of a few clones showed that the adenovirus titer was very low in the absence of coumermycin. Finally, the virus progeny was measured in duplicate by flow cytometry in the 10 best subclones. The combined results of the first and second screenings are shown in Figure 2. Only two clones (80 and 174) had comparable titers with the HEK293A-CymR-PS pool.

**Figure 3:**
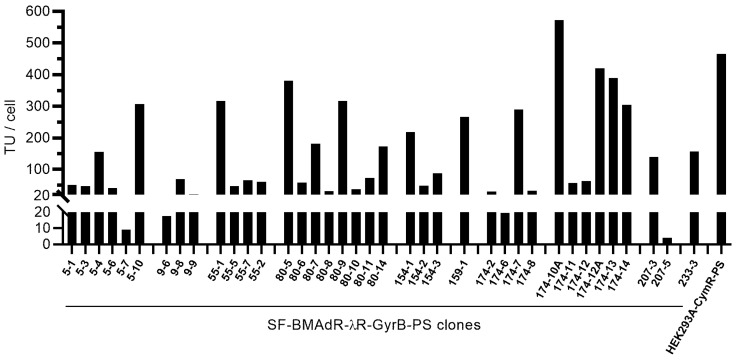
Production of AdPS-/CU by SF-BMAdR-λR-GyrB-PS subclones. Cells were infected using a MOI of 5 transduction unit(s) (TU)/cell in the presence of 5 nM of coumermycin. The specific productivities of infectious vector particles (TU/cell) were determined by flow cytometry (*n* = 1). The HEK293A-CymR-PS cell line was infected in the same manner in the presence of 50 μg/mL of cumate and used as a comparison.

To ensure that the clones were monoclonal, the 10 best clones were subcloned in 96-well plates and the 54 resulting subclones were expanded and analyzed by a cell imaging system to select for the highest GFP expression following infection with AdPS-/CU. In 38 of the highest GFP-expressing subclones, virus progeny was measured by flow cytometry (Figure 3).

**Figure 4:**
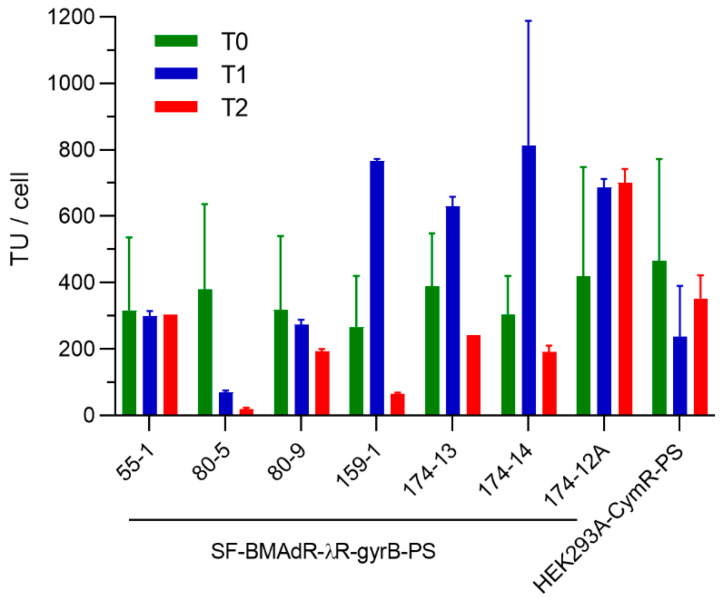
Stability of SF-BMAdR-λR-GyrB-PS subclones and the HEK293A-CymR-PS pool over a period of two months. A total of 500,000 cells were infected in 24 wells in duplicate (*n* = 2) with AdPS-/CU at a MOI of 5 TU/cell in the presence of coumermycin (5 nM) for SF-BMAdR-λGyrB-PS subclones and cumate for the HEK293A-CymR-PS pool. The specific productivities of infectious vector particles (TU/cell) were determined by flow cytometry; the mean ± SEM is shown.

As the continuous presence of antibiotics in the cell culture medium is not recommended, the stability of subclones over a period of two months in the absence of antibiotic selection was evaluated. The seven best subclones of SF-BMAdR-λGyrB-PS were cultured in the absence of antibiotics. The stability of the HEK293A-CymR-PS pool was also evaluated in parallel. At zero (one week after thawing), one, and two months, the progeny of AdPS-/CU in each stable cell line was measured in duplicate (Figure 4).

Based on these stability results, SF-BMAdR-λR-GyrB-PS-174-12A was selected as the best suspension cells for the production of PS-deleted AdV. The SF-BMAdR-λR-GyrB-PS-174-12A subclone produced 400–700 TU/cell of AdPS-/CU when tested for stability over a span of two months, which was even higher than the adherent HEK293A-CymR-PS.

### 3.3. Production and Purification of SC-AdVs in the SF-BMAdR-λR-GyrB-PS-174-12A Cell Line at a Larger Scale

To produce enough material for our ongoing research projects with SC-AdVs, production using the SF-BMAdR-λR-GyrB-PS-174-12A subclone at a larger scale was initiated before we fully completed characterizing and optimizing this cell line. Overall, 6 different SC-AdVs (a total of 8 lots) at a scale of 2 to 8.2 L were produced and purified (Table 2). Three lots of AdPS-/CU were produced to show the reproducibility. The viral particles (VP)/ml of AdPS-/CU varied by only two-fold between these three production and purification sets. The VP titers and infectious particles of the other viruses varied depending on the virus backbone and GOI.

### 3.4. Characterization of SF-BMAdR-λR-GyrB-PS-174-12A

The clones and subclones of SF-BMAdR-λR-GyrB-PS were selected based on the complementation of these cell lines for the production of AdPS-/CU. The CU gene is a highly toxic gene and its expression during virus production is prevented by constitutive expression of the repressor (CymR). However, in the OFF condition (absence of cumate), there is a basal level expression of CU that affects virus progeny. The specific productivity of AdPS-/CU in SF-BMAdR-λR-GyrB-PS subclones, at best, was a few hundred TU/mL. As the majority of transgenes are non-toxic to cells, we also studied the efficacy of the SF-BMAdR-λR-GyrB-PS-174-12A subclone in the production of a PS-deleted AdV not carrying a toxic transgene. SF-BMAdR-λR-GyrB-PS-174-12A and HEK293A-CymR were infected with AdPS- and the specific productivity was determined by a TCID_50_ assay. The SF-BMAdR-λR-GyrB-PS-174-12A subclone produced, on average, 3417 infectious particles compared to 4066 for the HEK293A-CymR pool (Figure S2).

**Figure 5:**
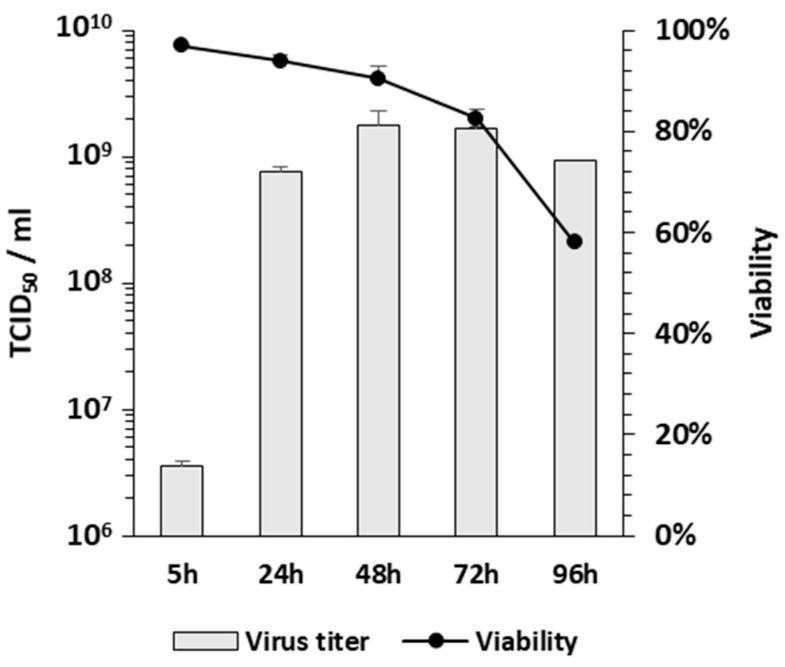
Virus growth curve of AdPS- in the SF-BMAdR-λR-GyrB-PS-174-12A subclone. Two 125 mL shaker flasks of cells at 25 mL of working volume were infected with a MOI of 5 TCID_50_ in the presence of 5 nM of coumermycin (*n* = 3). The cell viability was recorded at each time point and the virus progeny was measured by a TCID_50_ assay in duplicate, expressed as TCID_50_/mL, and shown as mean ± SEM.

Determining the best harvesting time is critical for the large-scale production of AdV. On the first day of infection, we demonstrated that different backbones of PS-deleted AdV showed delays in the expression of the transgene compared to RC-AdV. However, transgene expression reached the same level in both groups at 48 hpi and declined at 72 hpi [34]. To determine the best harvesting time to allow maximum production, we evaluated the AdPS- growth curve in the SF-BMAdR-λR-GyrB-PS-174-12A subclone from 5 h to 96 hpi. The best harvesting time was between 48 and 72 hpi, with cell viability between 91 and 83% (Figure 5), which is similar to the RD-AdV production in our laboratory.

The doubling time of the SF-BMAdR-λR-GyrB-PS-174-12A subclone was compared with its parental cell line. The doubling time for SF-BMAdR-λR-GyrB-PS-174-12A is 43.53 h in the Pro293s culture medium, which is slightly longer compared to 31–33 h of its parental cell line [26].

The screening of clones and subclones for the SF-BMAdR-λR-gyrB-PS cell line, thus far, was performed using the PRO293s medium. As the choice of culture medium during the infection period influences the virus yield, we evaluated the production of AdPS/CU by SF-BMAdR-λR-GyrB-PS-174-12A cells cultured in three different types of culture media, i.e., Pro293s, Balance CD HEK293 (Irvine Scientific), and FreeStyle 293 (Thermo Fisher Scientific, Waltham, MA, USA). Specific productivity was the highest in PRO293s and the lowest in FreeStype 293 (Figure S3).

**Figure 6:**
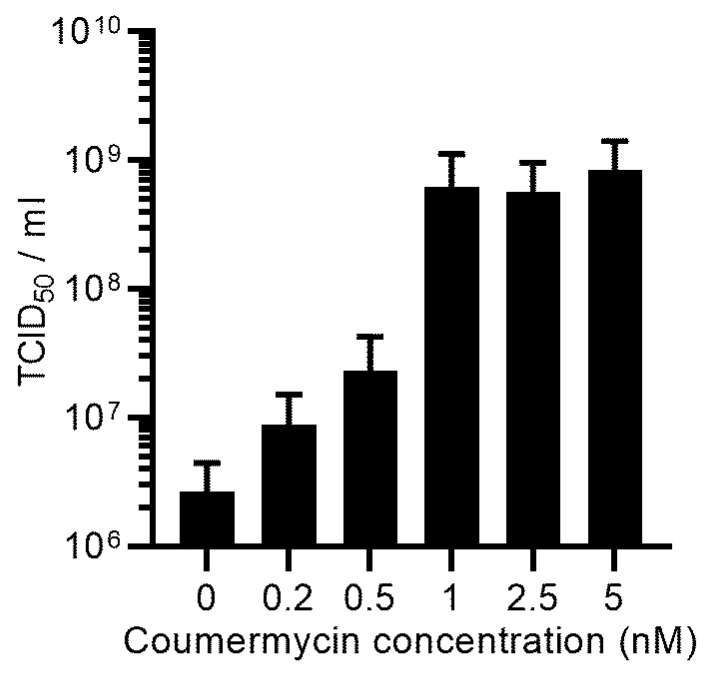
The infected SF-BMAdR-λR-GyrB-PS-174-12A cells with AdPS- (*n* = 2) were induced with different concentrations of coumermycin at 5 hpi. The virus progeny was measured by the TCID_50_ assay in duplicate, expressed as TCID_50_/mL, and shown as mean ± SEM.

The expression of the PS gene in the SF-BMAdR-λR-GyrB-PS cell line was under the control of the coumermycin switch. We used coumermycin at a concentration of 5 nM, as suggested by Zhao et al. [24] for the expression of Luciferase. However, as the overexpression of PS could affect virus progeny, we evaluated AdPS- production in partially induced conditions to find the optimal PS expression level to achieve the highest AdPS- yield possible. A concentration as low as 1 nM fully induced the expression of the PS gene and there was no significant difference between doses of 1 and 5 nM (Figure 6). However, at suboptimal doses of 0.5 and 0.2 nM, the titer dropped 33 and 92 times, respectively, compared to a concentration of 5 nM. At the basal level (the absence of coumermycin) the titer of virus progeny dropped more than 300 times. These data confirmed the fact that a dose of 5 nM of coumermycin was appropriate and had no negative impact on virus production.

## 4. Discussion

The objective of this study was to improve the titer of the protease-deleted adenovirus in adherent and suspension cultures and eliminate the use of FBS during the production scale-up. The best clone from our previous adherent cell line, HEK293A-PS-CB6, in which the PS gene was under the control of a heat shock promoter [25], produced 25 times less virus than the pool of HEK293A-CymR-PS. Another advantage was that the experimental times in performing the plaque assay and the TCID_50_ assay were shortened due to the faster cytopathic effect appearances in the HEK293A-CymR-PS cell line compared with the HEK293A-PS-CB6 cell line. These two improvements were sufficient for the easy generation and quantification of PS-deleted AdV without the need to further clone the pool.

At a small scale, the titer of AdPS- was 9.4 times higher in SF-BMAdR-λR-GyrB-PS-174-12A compared with 293rTA-PS#7. This significant increase in the virus titer demonstrated the high productivity and robustness of our SC-RAdV platform. Moreover, the SF-BMAdR-λR-GyrB-PS cell line grows in suspension culture in the absence of FBS contrary to 293rTA-PS cell lines, which need the presence of 5% FBS.

In the current study, we did not measure the copy number of the PS gene integrated into each stable cell line or analyze the PS expression by Western blot as a screening method. It is logical to speculate that after each transduction, the copy number will increase depending on the original titer of the lentivirus stock used for transduction. However, we have already shown that increasing the level of the PS expression does not guarantee a higher titer and, thus, high levels of the PS expression would not have been useful criteria to identify the best clones since the optimal range for the PS expression level is quite narrow [9,25,29]. Our previous data showed that overexpression or the early expression of the PS gene is deleterious for virus progeny. Complementation of a PS-deleted AdV was the best in 293rtTA-PS#7 and 293tTA-PS#15 clones under non-induced conditions. Under these conditions, the level of PS expressed by the cells was too low to be detectable in Western blot [9]. Thus, the quantification of infectious viral particles generated by each cell line is the best indicator for the selection of the best pool, clone, or subclone.

Previously, we demonstrated that the replication-defective AdV-expressing CU gene under the control of the CMV5CuO promoter (Ad/CU) had a similar titer as AdP-/CU in the OFF condition in SF-BMAdR-λR-GyrB-PS (when the expression of CU was suppressed by the expression of CymR). The Ad/CU titer was decreased five times in a non-inducible cell line (absence of CymR) when the expression of CU was not prevented [26]. These data confirm that for the production of AdVs expressing a toxic protein (such as CU or PS), an inducible promoter should be used to increase the titer. A wild type AdV without any deletion could produce up to 26,000 infectious particles per cell in HEK293A [34]. Our best stable cell line produced about 4000 infectious particles per cell. The lower titer may have been caused by the inadequate expression (amount and/or timing) of PS by the complementary cell line. The titer of AdPS-/CU in SF-BMAdR-λR-GyrB-PS-174-12A, when induced for PS production, is similar to the titer of the PS-intact version of this virus in the parental SF-BMAdR cell line. In general, in the case of RD-AdV with E1 deletion, the complementary cell line (HEK293A) could not completely rescue the viral production yield and we observed that the yield is usually 2–3 times lower compared to RC-AdV.

The BMAdR-SF-CymR-PS pool was chosen over 293S-CymR-PS in preventing the formation of RCA. When choosing between the two BMAdR cell lines, even though SF-BMAdR-λR-GyrB-PS#174-12A had a lower titer of AdPS- compared to the SF-BMAdR-CymR-PS#27 cell line, our selected suspension cell line was SF-BMAdR-λR-GyrB-PS#174-12A since its ON/OFF ratio value was 52 times higher than the SF-BMAdR-CymR-PS#27 cell line. Moreover, this cell line was produced by transfection, contrary to SF-BMAdR-CymR-PS#27, which would most likely facilitate its approbation by regulatory agencies for the production of material for human applications.

Scalability and reproducibility of the SF-BMAdR-λR-GyrB-PS-174-12A subclone for the production of six different SC-AdVs were evaluated by up to 8.2 L before the final characterization of the cell line and process optimization. Some lots were prepared for our ongoing projects to compare these viruses with RD-AdVs. Consequently, the infectious titers of the purified viruses were measured using the “Adeno-X™ Rapid Titer Kit” because the HEK293A cell lines could be used with this method for both SC-AdV and RD-AdV quantification, contrary to the TCID_50_ assay and plaque purification, which require HEK293A-CymR-PS and HEK293A, respectively. However, the Adeno-X^TM^ Rapid titer is very time-consuming and could not be applied to quantify the large number of AdVs in the clones and subclones. TCID_50_ was used to screen the pool and clones by APS- (which does not have a marker) and flow cytometry was used to screen the clone and subclones of AdPS-/CU expressing GFP. At a larger scale of production, coumermycin was used at a concentration of 20 nM. This higher concentration had no negative impact on the virus titer as our preliminary results showed a concentration of up to 50 nM can be used without reducing the virus titer.

The VP/ifu ratio of SC-AdVs after purification is higher than what is observed for the first-generation AdV (ratio of 10 to 20). A higher ratio of VP/ifu suggests that most virions are non-infectious. The inadequate expression of the PS gene or the wrong expressing timing could be the reason as PS is essential for the maturation and assembly of viral proteins, as well as for the release of virions from infected cells. Furthermore, viral entry into host cells requires that the PS be packaged within the mature virion [9]. The infectious titers of three AdPS-/CU lots were lower than the titer of this virus obtained during screening and stability studies. This difference in the titer could be explained by the two quantification methods that were used. The Adeno-X™ Rapid titration method (which was used for the larger production) underestimates the viral titer by a factor of 2 compared with TCID_50_ [35]. Moreover, there is loss during the purification process. The infectious titer of AdPS-/CU lot #2 before purification was measured to confirm this hypothesis. The ifu/mL of the non-purified stock was 362 compared to 134 after purification. This results in a hypothetical titer of 724 TCID50/ml that is in the same range as the small-scale production. The titers for total viral and infectious particles for the other SC-AdVs varied depending on the adenovirus backbone and, most importantly, on the expressed transgene.

## 5. Conclusions

The SC-AdVs, such as those based on the deletion of the PS gene, are promising viral vectors for the development of potent vaccines and the treatment of cancer [10,11,12]. Recently, we demonstrated that the expression of a GOI between the right-inverted terminal repeat and the E4 region of the adenovirus in a wide variety of PS-deleted adenovirus vectors is as efficient as the PS+ counterpart [34]. However, it is essential to facilitate the development and translation of our PS-deleted AdV toward clinical applications and appropriate cell lines for the large-scale production and quantification of these types of viruses. Different stable cell lineages were, therefore, generated using either lentivirus or standard transfection methods. The best adherent cell pool that we developed, HEK293A-CymR-PS, is suitable for generating the recombinant protease-deleted AdV by recombination or transfection. It could also be used for plaque purification and quantification of PS-deleted AdV. On the other hand, the SF-BMAdR-λR-GyrB-PS-174-12A subclone, which was adapted to suspension culture using medium without animal-derived components, can be used for producing PS-deleted AdV at small and large scales with comparable titers to RD-AdV.

## Figures and Tables

**Table 1:** Stable cell pools generated by lentiviruses. The parental cell lines were transduced 2 to 5 times with different lentiviruses expressing the PS gene or repressor CymR, as indicated. The production of AdPS- was measured in each pool by the TCID_50_ assay in duplicate and specific productivity was expressed as the TCID_50_/cell. The average of the TCID_50_ assay titer is presented. Only one pool in each category with the highest titer was identified with the indicated designation and used for subsequent experiments. Numbers in the 2nd to 4th columns represent the number of transfections with each lentivirus.

Parental Cell Line	Lenti-CMV5-Cuo-PS	Lenti-CR5-PS	Lenti-CymR	New Stable Cell Pool	Cumate for PS Production	Sample Size	Average of AdPS- Titer (TCID50/Cell)
HEK293A (adherent)	1×			HEK293A-CymR-PS	YES	3	5730
	2×				YES	2	1054
HEK293A-cTA (adherent)		1×			NO	2	618
		2×		HEK293A-cTA-PS	NO	2	2640
293S (Gibco) (suspension)	1×				YES	2	85
	1×		1×		YES	2	95
	1×		2×		YES	2	300
	2×		2×		YES	1	641
	3×		2×	293S-CymR-PS	YES	1	4335
BMAdR-SF (suspension)	1×				YES	2	113
	1×		1×		YES	2	43
	1×		2×		YES	2	471
	2×		2×		YES	1	2028
	3×		2×	BMAdR-SF-CymR-PS	YES	1	3000

**Table 2:** Production and purification scale-up of SC-AdVs. Overall, 8 lots of SC-AdVs were produced in the SF-BMAdR-λR-GyrB-PS-174-12A cell line. The viruses were purified and the infectious particles were quantified after purification.

Characteristics of Viruses				Production Phase			Purification Phase				
Virus Name	Virus Backbone	Expression Cassette	Reference	Total Cells (Infection Time)	Volume (mL)	Viable Cells/mL	Total Viral Particles	Total Infectious Particles	VP/Ifu ratio	VP/Cell	ifu/Cell
AdPS-/CU (lot# 2) *	**PS, E1, and E3 deleted**	**CD::UPRT fusion gene plus GFP**	**[10]**	**1.37 × 10^9^**	**2500**	**5.48 × 10^5^**	**2.04 × 10^13^**	**1.84 × 10^11^**	**111**	**14,891**	**134**
AdPS-/CU (lot# 3) *	**PS, E1, and E3 deleted**	**CD::UPRT fusion gene plus GFP**	**[10]**	**1.35 × 10^9^**	**2500**	**5.40 × 10^5^**	**9.82 × 10^12^**	**1.07 × 10^11^**	**92**	**7271**	**79**
AdPS-/CU (lot# 4) *	**PS, E1, and E3 deleted**	**CD::UPRT fusion gene plus GFP**	**[10]**	**1.00 × 10^9^**	**2000**	**5.00 × 10^5^**	**1.13 × 10^13^**	**2.29 × 10^11^**	**49**	**11,276**	**229**
AdPS- (lot# 2) *	**PS deleted**	**non**	**[9]**	**1.00 × 10^9^**	**2000**	**5.00 × 10^5^**	**1.89 × 10^13^**	**ND**	**ND**	**18,880**	**ND**
**AdPS-/HCV-NS3-5a**	**PS, E1, E3 and E4 (except ORF6) deleted**	**NS3-5a of HCV and E1a of adenovirus**	**[11]**	**1.00 × 10^9^**	**2000**	**5.00 × 10^5^**	**1.49 × 10^12^**	**3.88 × 10^10^**	**39**	**1494**	**39**
**AdPS-/Her2**	**PS, E1, E3 deleted**	**Rat HER2/neu gene and E1a of adenovirus**	**[12]**	**4.01 × 10^9^**	**8185**	**4.90 × 10^5^**	**4.78 × 10^13^**	**1.62 × 10^12^**	**30**	**11,917**	**404**
**AdPS-/TRP2-IRES-GM_CSF**	**PS, and E4 (except ORF6) deleted**	**TRP2 and GMCSF**	**unpublished data**	**1.50 × 10^9^**	**3000**	**5.00 × 10^5^**	**4.12 × 10^13^**	**1.89 × 10^12^**	**22**	**27,459**	**1260**
**AdPS-/GP100-IRES-GM_CsF**	**PS, and E4 (except ORF6) deleted**	**GP100 and GMCSF**	**unpublished data**	**1.50 × 10^9^**	**2760**	**5.43 × 10^5^**	**4.63 × 10^13^**	**1.48 × 10^12^**	**31**	**30,880**	**987**

* These lots were not used in these publications, but the publications describe the adenovirus.

## Data Availability

The data presented in this study are available in the current article or Supplementary Materials. The raw data or unpublished data that support the findings of this study are available upon request from the corresponding author.

## Supplementary Materials

The following supporting information can be downloaded at https://www.mdpi.com/article/10.3390/cells12040619/s1, Figure S1: Production of AdPS- by SF-BMAdR-CymR-PS pool and clones with cumate regulation. Figure S2: Comparative production of AdPS- by suspension SF-BMAdR-λR-GyrB-PS-174-12A subclone and adherent HEK29A-CymR-PS pool. Figure S3: Effect of media in virus yield.
