# Supplementary material for: Complementary Cell Lines for Protease Gene-Deleted Single-Cycle Adenovirus Vectors
Source: Cells. 2023 Feb 14;12(4):619. doi: 10.3390/cells12040619 (PMC9954690; doi:10.3390/cells12040619)
Supplement: Supplementary file 1 [file cells-12-00619-s001.zip › cells-2128465-supplementary.pdf]

Supplementary Materials

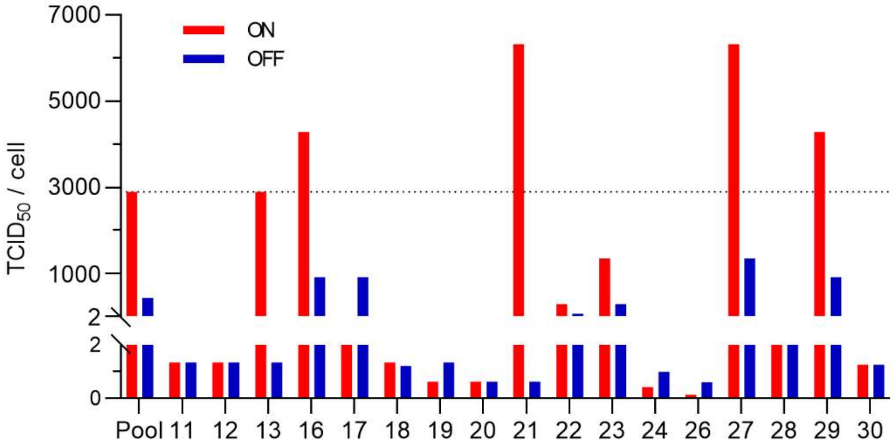

Figure S1. Production of AdPS- by SF-BMAdR-CymR-PS pool and clones with cumate regulation. Cells were infected with AdPS- using an MOI of 5 TCID50 / cells in the presence or absence of cumate (n = 1). The virus yield was measured by TCID50 and expressed as TCID50/ cell.

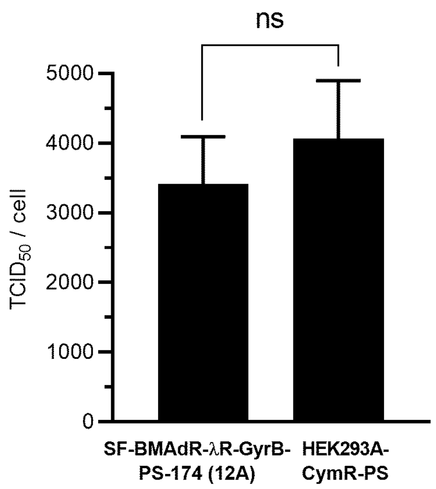

Figure S2. Comparative production of AdPS- by suspension SF-BMAdR-λR-GyrB-PS-174-12A subclone and adherent HEK29A-CymR-PS pool. Three wells of 6-well plate (n = 3) at a concentration of 500,000 cells /ml were infected with AdPS- with an MOI of 5 TCID50. For comparison purposes, wells of 6-well plate of HEK293A-CymR-PS at a concentration of 1,000,000 cells/well also were infected. Coumermycin was added at a concentration of 5 nM to SF-BMAdR-λR-GyrB-PS-174-12A and cumate was added at a concentration of 50 μg/ ml to HEK293A-CymR-PS to induce the PS production in both cell lines. The specific productivity for each sample was determined by TCID50 assay at 48 hpi in duplicate and shown as mean ± SEM.

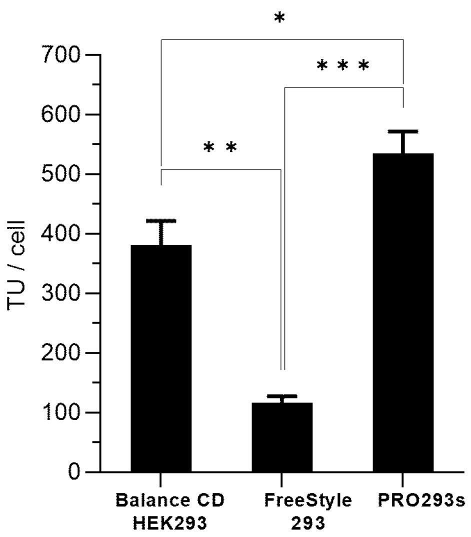

Figure S3. Effect of media in virus yield. Three ml of SF-BMAdR-λR-GyrB-PS-174-12A cells (cultured in three different media) at a concentration of 500,000 cells/ml were infected with AdPS-/CU with MOI of 5 TU/ cell in 6-well plate (n = 3). Two days post infection and after three freeze/thaw cycles, the titer was measured by flow cytometry and expressed as TU/ cell and shown as mean ± SEM.
